# Supplementary material for: Modeling glioblastoma heterogeneity as a dynamic network of cell states
Source: Mol Syst Biol. 2021 Sep 16;17(9):e10105. doi: 10.15252/msb.202010105 (PMC8444284; doi:10.15252/msb.202010105)
Supplement: Supplementary file 5 — Source Data for Figure 3 [file MSB-17-e10105-s001.zip › Figure3A_sourcedata/GSEA_3065/nosigdb_state5.GseaPreranked.1622718435914/LARSSON_STATE_3.html]

Details for gene set LARSSON\_STATE\_3[GSEA]

|  || Dataset | state5 |
| Phenotype | NoPhenotypeAvailable |
| Upregulated in class | na\_pos |
| GeneSet | LARSSON\_STATE\_3 |
| Enrichment Score (ES) | 0.9026765 |
| Normalized Enrichment Score (NES) | 3.6329522 |
| Nominal p-value | 0.0 |
| FDR q-value | 0.0 |
| FWER p-Value | 0.0 |
Table: GSEA Results Summary

  

Fig 1: Enrichment plot: LARSSON\_STATE\_3      
 Profile of the Running ES Score & Positions of GeneSet Members on the Rank Ordered List

  

| PROBE | GENE SYMBOL | GENE\_TITLE | RANK IN GENE LIST | RANK METRIC SCORE | RUNNING ES | CORE ENRICHMENT || 1 | MALAT1 |  |  | 0 | 1.196 | 0.0274 | Yes |
| 2 | KCNQ1OT1 |  |  | 8 | 0.931 | 0.0480 | Yes |
| 3 | PTPRZ1 |  |  | 12 | 0.862 | 0.0674 | Yes |
| 4 | CD9 |  |  | 14 | 0.857 | 0.0869 | Yes |
| 5 | BCAN |  |  | 15 | 0.850 | 0.1064 | Yes |
| 6 | MTRNR2L12 |  |  | 18 | 0.825 | 0.1251 | Yes |
| 7 | PTN |  |  | 19 | 0.824 | 0.1440 | Yes |
| 8 | MDM4 |  |  | 21 | 0.811 | 0.1625 | Yes |
| 9 | CD63 |  |  | 24 | 0.769 | 0.1799 | Yes |
| 10 | SOX6 |  |  | 28 | 0.754 | 0.1969 | Yes |
| 11 | PDIA6 |  |  | 29 | 0.742 | 0.2139 | Yes |
| 12 | APLP2 |  |  | 31 | 0.733 | 0.2306 | Yes |
| 13 | GRIA2 |  |  | 32 | 0.732 | 0.2474 | Yes |
| 14 | HSP90B1 |  |  | 33 | 0.729 | 0.2641 | Yes |
| 15 | SCRG1 |  |  | 36 | 0.715 | 0.2802 | Yes |
| 16 | PLAT |  |  | 37 | 0.714 | 0.2966 | Yes |
| 17 | APP |  |  | 38 | 0.712 | 0.3129 | Yes |
| 18 | SQLE |  |  | 39 | 0.705 | 0.3291 | Yes |
| 19 | HSPA5 |  |  | 43 | 0.698 | 0.3448 | Yes |
| 20 | SPCS2 |  |  | 44 | 0.689 | 0.3606 | Yes |
| 21 | FUS |  |  | 47 | 0.665 | 0.3756 | Yes |
| 22 | PDIA3 |  |  | 49 | 0.652 | 0.3904 | Yes |
| 23 | PGRMC1 |  |  | 52 | 0.639 | 0.4048 | Yes |
| 24 | CANX |  |  | 53 | 0.634 | 0.4194 | Yes |
| 25 | CALR |  |  | 55 | 0.631 | 0.4337 | Yes |
| 26 | LRPAP1 |  |  | 57 | 0.619 | 0.4478 | Yes |
| 27 | GPM6A |  |  | 60 | 0.609 | 0.4615 | Yes |
| 28 | B2M |  |  | 61 | 0.601 | 0.4753 | Yes |
| 29 | ATP6AP2 |  |  | 62 | 0.597 | 0.4890 | Yes |
| 30 | TMBIM6 |  |  | 68 | 0.584 | 0.5018 | Yes |
| 31 | NCAM1 |  |  | 70 | 0.581 | 0.5150 | Yes |
| 32 | SSR4 |  |  | 72 | 0.573 | 0.5281 | Yes |
| 33 | CALU |  |  | 78 | 0.563 | 0.5404 | Yes |
| 34 | HMGCR |  |  | 79 | 0.562 | 0.5533 | Yes |
| 35 | PRDX4 |  |  | 80 | 0.553 | 0.5660 | Yes |
| 36 | PON2 |  |  | 84 | 0.546 | 0.5782 | Yes |
| 37 | FGFBP3 |  |  | 85 | 0.545 | 0.5907 | Yes |
| 38 | HACD3 |  |  | 91 | 0.542 | 0.6025 | Yes |
| 39 | LMAN1 |  |  | 95 | 0.533 | 0.6144 | Yes |
| 40 | LAPTM4B |  |  | 97 | 0.532 | 0.6265 | Yes |
| 41 | GPC1 |  |  | 98 | 0.530 | 0.6387 | Yes |
| 42 | DSEL |  |  | 107 | 0.518 | 0.6496 | Yes |
| 43 | TIMP1 |  |  | 109 | 0.518 | 0.6614 | Yes |
| 44 | WLS |  |  | 116 | 0.509 | 0.6724 | Yes |
| 45 | ITM2B |  |  | 117 | 0.509 | 0.6841 | Yes |
| 46 | TMED9 |  |  | 119 | 0.508 | 0.6956 | Yes |
| 47 | STT3B |  |  | 120 | 0.508 | 0.7072 | Yes |
| 48 | RTN3 |  |  | 123 | 0.505 | 0.7186 | Yes |
| 49 | TFPI2 |  |  | 124 | 0.504 | 0.7301 | Yes |
| 50 | MANF |  |  | 130 | 0.499 | 0.7410 | Yes |
| 51 | BSG |  |  | 135 | 0.493 | 0.7519 | Yes |
| 52 | CCDC47 |  |  | 140 | 0.490 | 0.7626 | Yes |
| 53 | EMC7 |  |  | 146 | 0.487 | 0.7732 | Yes |
| 54 | FAT1 |  |  | 155 | 0.478 | 0.7833 | Yes |
| 55 | DYNC1H1 |  |  | 160 | 0.475 | 0.7937 | Yes |
| 56 | ABHD2 |  |  | 163 | 0.473 | 0.8044 | Yes |
| 57 | MMP16 |  |  | 165 | 0.470 | 0.8150 | Yes |
| 58 | SPCS3 |  |  | 183 | 0.454 | 0.8235 | Yes |
| 59 | GGH |  |  | 184 | 0.453 | 0.8339 | Yes |
| 60 | SLC44A1 |  |  | 216 | 0.428 | 0.8402 | Yes |
| 61 | ALCAM |  |  | 225 | 0.422 | 0.8490 | Yes |
| 62 | RPN2 |  |  | 244 | 0.411 | 0.8564 | Yes |
| 63 | EMP3 |  |  | 264 | 0.400 | 0.8635 | Yes |
| 64 | PIF1 |  |  | 266 | 0.400 | 0.8725 | Yes |
| 65 | GPNMB |  |  | 281 | 0.393 | 0.8799 | Yes |
| 66 | DST |  |  | 313 | 0.376 | 0.8851 | Yes |
| 67 | POSTN |  |  | 316 | 0.375 | 0.8934 | Yes |
| 68 | TM9SF3 |  |  | 339 | 0.364 | 0.8993 | Yes |
| 69 | LAMC1 |  |  | 382 | 0.348 | 0.9026 | Yes |
| 70 | CD320 |  |  | 456 | 0.324 | 0.9018 | Yes |
| 71 | PLD5 |  |  | 540 | 0.300 | 0.8994 | Yes |
| 72 | VGF |  |  | 571 | 0.292 | 0.9027 | Yes |
| 73 | LPL |  |  | 646 | 0.276 | 0.9007 | No |
| 74 | ASPM |  |  | 980 | 0.213 | 0.8682 | No |
| 75 | PRKDC |  |  | 1051 | 0.204 | 0.8650 | No |
| 76 | NASP |  |  | 1225 | 0.182 | 0.8497 | No |
| 77 | CENPF |  |  | 1531 | 0.149 | 0.8188 | No |
| 78 | SPTBN1 |  |  | 1880 | 0.120 | 0.7825 | No |
| 79 | HNRNPU |  |  | 2204 | 0.098 | 0.7484 | No |
| 80 | SYNE2 |  |  | 2881 | 0.058 | 0.6738 | No |
| 81 | IQGAP1 |  |  | 3424 | 0.031 | 0.6136 | No |
| 82 | TOP2A |  |  | 5722 | -0.063 | 0.3569 | No |
| 83 | MYH10 |  |  | 5961 | -0.072 | 0.3318 | No |
| 84 | MKI67 |  |  | 6211 | -0.086 | 0.3058 | No |
| 85 | HSP90AB1 |  |  | 6611 | -0.109 | 0.2635 | No |
| 86 | HSP90AA1 |  |  | 6962 | -0.134 | 0.2272 | No |
Table: GSEA details [plain text format]

  

Fig 2: LARSSON\_STATE\_3: Random ES distribution      
 Gene set null distribution of ES for **LARSSON\_STATE\_3**
